# Metabolic Fingerprinting of *Pseudomonas putida* DOT-T1E Strains: Understanding the Influence of Divalent Cations in Adaptation Mechanisms Following Exposure to Toluene

**DOI:** 10.3390/metabo6020014

**Published:** 2016-04-26

**Authors:** Ali Sayqal, Yun Xu, Drupad K. Trivedi, Najla AlMasoud, David I. Ellis, Royston Goodacre

**Affiliations:** Manchester Institute of Biotechnology, School of Chemistry, The University of Manchester, Manchester M1 7DN, UK; ali.sayqal@postgrad.manchester.ac.uk (A.S.); Yun.Xu-2@manchester.ac.uk (Y.X.); drupad.trivedi@manchester.ac.uk (D.K.T.); nagla.almasoud-2@postgrad.manchester.ac.uk (N.A.); D.Ellis@manchester.ac.uk (D.I.E.)

**Keywords:** fingerprinting, efflux pumps, *P. putida* DOT-T1E, toluene, stress tolerance, LPS, Mg^2+^, Ca^2+^, FT-IR

## Abstract

*Pseudomonas putida* strains can adapt and overcome the activity of toxic organic solvents by the employment of several resistant mechanisms including efflux pumps and modification to lipopolysaccharides (LPS) in their membranes. Divalent cations such as magnesium and calcium play a crucial role in the development of solvent tolerance in bacterial cells. Here, we have used Fourier transform infrared (FT-IR) spectroscopy directly on cells (metabolic fingerprinting) to monitor bacterial response to the absence and presence of toluene, along with the influence of divalent cations present in the growth media. Multivariate analysis of the data using principal component-discriminant function analysis (PC-DFA) showed trends in scores plots, illustrating phenotypic alterations related to the effect of Mg^2+^, Ca^2+^ and toluene on cultures. Inspection of PC-DFA loadings plots revealed that several IR spectral regions including lipids, proteins and polysaccharides contribute to the separation in PC-DFA space, thereby indicating large phenotypic response to toluene and these cations. Finally, the saturated fatty acid ratio from the FT-IR spectra showed that upon toluene exposure, the saturated fatty acid ratio was reduced, while it increased in the presence of divalent cations. This study clearly demonstrates that the combination of metabolic fingerprinting with appropriate chemometric analysis can result in practicable knowledge on the responses of important environmental bacteria to external stress from pollutants such as highly toxic organic solvents, and indicates that these changes are manifest in the bacterial cell membrane. Finally, we demonstrate that divalent cations improve solvent tolerance in *P. putida* DOT‑T1E strains.

## 1. Introduction

Organic solvents such as benzene, toluene, styrene and xylenes are known to be highly toxic to microorganisms, as these aromatic solvents are known to partition and preferentially accumulate in the bacterial cell membrane, thereby disorganising its structure and impairing cell membrane integrity and function, ultimately leading to cell death [1,2,3,4]. Nevertheless, it has been reported that some microorganisms have the ability to assimilate these toxic organic solvents even when the solvent concentration is very high. In 1989, the first report of an organic solvent-resistant bacterium, resistant to high toxic levels of solvent, was observed [1]. Inoue and Horikoshi isolated a strain of *Pseudomonas putida* (strain HI-2000) which was able to grow in the presence of 50% (*v*/*v*) toluene. This surprising finding has since been confirmed by others [5,6,7,8,9], and the search has begun in earnest to discover the mechanisms behind this solvent tolerance.

Bacteria can defend themselves from the action of organic solvents by various adaptation mechanisms. Several studies have suggested that efflux pumps [10,11], divalent ions, such as magnesium ions [8,12], and the order organisation of cell surface lipopolysaccharides [13] contribute to solvent tolerance. In *P. putida* DOT-T1E, although high solvent tolerance is acquired mainly by the presence of efflux pumps [11,14], various other mechanisms contribute to organic solvent tolerance as well [15].

Solvent-tolerant microorganisms play an important role in several biotechnological applications and areas such as bioremediation, agriculture and biocatalysis [16,17,18,19]. Bioremediation involves the employment of microorganisms to convert toxic chemicals found in the environment into benign or less toxic species of chemicals [20,21,22]. Whole-cell biocatalysis involves the production of specialty or fine chemicals, and often employs two-phase systems in order to extract and reduce the concentration of toxic products (or indeed substrates) from the aqueous phase [23,24]. This would decrease the deleterious effects of any toxic products and hence the biocatalyst remains active, making product recovery easier and less costly [25,26]. Solvent tolerant microorganisms are a growing field of study in biotechnological applications, and more in-depth knowledge to aid in the understanding of the mechanisms of solvent tolerance is required. Researchers have suggested that genetic engineering, pre-exposure of bacterial cultures to low concentrations of toxic solvent, and magnesium ions contribute to the enhancement of solvent tolerance [4,8,27,28]. One study investigated the effect of various metal ions such as Mg^2+^, Ca^2+^, Pb^2+^ and W^6+^ on the stabilization of toluene tolerance of *P. putida* IH-2000, and it was found that among the ions examined, Mg^2+^ and Ca^2+^ were the most effective in stabilization of toluene tolerance, thereby suggesting that metal ions may enhance solvent tolerance in living cells [12].

Metabolomics covers the identification and quantification of the metabolome (small molecules involved in cellular metabolic processes) employing different analytical techniques [29,30,31,32]. One of the core high-throughput approaches within the expanding field of metabolomics is metabolic fingerprinting [33]. With this approach, a rapid biochemical snapshot is obtained from cells, tissue, or biofluids that have been perturbed and any changes detected and correlated with fingerprints from “normal” or typical control samples. Therefore, metabolic fingerprinting can be considered as a rapid, global, high-throughput approach to provide sample provenance (classification), which can also be utilized as a screening tool to differentiate and classify samples quickly from different biological status or origin [33]. Metabolic fingerprinting also normally entails minimal sample preparation and can be undertaken via one of a number of technologies, here, we used FT-IR spectroscopy.

FT-IR spectroscopy allows for a very rapid, high-throughput and non-destructive analysis of a broad range of sample types, and has been shown to be a valuable tool for the characterization of cultured bacteria [34,35,36,37]. Indeed, its application to the analysis of bacteria by Dieter Naumann and co-workers in the 1980s led to an explosion of activity in this area, and has subsequently continued to be applied to many others areas of research [38]. The technique involves the observation of vibrations of molecules following the interrogation of a sample with an infrared beam, and the resultant infrared absorbance spectrum represents a so-called “fingerprint” which is characteristic of any (bio)chemical substance [34,39,40].

The aim of this study was to elucidate whether divalent cations interact with efflux pumps or other resistant mechanisms to improve solvent tolerance in *P. putida* DOT-T1E strains, and to highlight the role of the analytical techniques to measure these microbial responses. We employed FT-IR spectroscopy to generate rapid and robust biochemical fingerprints of *P. putida* DOT-T1E strains: wild-type and mutants with impaired efflux pump activity. This analytical technique in combination with chemometrics can be used to observe metabolite changes that could be indicative of novel adaptation mechanisms, or support postulated adaptation mechanisms, and add to our knowledge in this important area of environmental microbiology.

## 2. Material and Methods

### 2.1. Bacterial Strains and Growth Conditions

The bacterial strains used in this study were *P. putida* DOT-T1E [8], *P. putida* DOT-T1E-PS28 (lacking the TtgGHI pump) [11] and *P. putida* DOT-T1E-18 (lacking the TtgABC pump) [27]. *P. putida* strains were routinely grown in nutrient agar plates to obtain fresh axenic cultures, which were then inoculated onto LB liquid medium and grown for 24 h at 30 °C with shaking (200 rpm) in an orbital incubator (Infors HT Ltd., Surrey, UK).

### 2.2. Growth in Response to Toluene in the Absence and Presence of Divalent Cations, Sample Collection and Analysis

The overnight cultures were diluted to an optical density at 660 nm (OD_660_) of 0.2 in 50 mL of fresh LB medium supplemented with or without 7 mM magnesium and 3 mM calcium in the absence and presence of 0.05% (*v*/*v*) toluene and grown overnight at 30 °C with shaking (200 rpm). All flask cultures were sealed with Suba-Seal to prevent toluene leakage.

#### 2.2.1. Growth Curve Monitoring

Bacterial cultures were monitored at various time points (1, 5, 10, 22, 30, 48 h) using a Biomate 5 (CarePlanTM, UK) at 660 nm (100 µL samples were measured) and the growth recorded as an increase or decrease in the turbidity of cultures during incubation.

#### 2.2.2. Analysis of Bacterial Cells by Fourier Transform Infrared (FT-IR) Spectroscopy

##### Sample Preparation

After 8 h incubation, cells had reached the mid-exponential phase, and 2 mL aliquots of *P. putida* cultures were harvested by centrifugation at 11,500 ×*g* for 5 min at 4 °C (ThermoFisher CR3.22, UK). Culture supernatant was discarded, and cells washed twice with 2 mL of physiological saline solution (0.9% NaCl). Cell pellets were stored at −80 °C until further required, and the procedure was conducted in triplicate.

Samples were defrosted on wet ice, suspended in saline solution and normalised according to OD at 660 nm. From the suspension, 20 µL aliquots were pipetted in triplicate onto a 96-well silicon FT-IR sampling plate (Bruker Optics, Banner Lane, Coventry, UK). Moisture was evaporated from the samples by drying the prepared plates in a desiccator at ambient temperature for 7 h. This step was applied to avoid strong water absorption in the mid-IR region.

##### Instrument Setup

Prepared sample plates were then loaded onto a motorised microplate module (HTS-XT™) [41], attached to an Equinox 55 infrared spectrometer (Bruker Optics, Banner Lane, Coventry, UK), equipped with a deuterated triglycine sulfate (DTGS) detector for transmission measurements of the sample to be acquired. Spectra were collected over the wavenumber range 4000–600 cm^−1^, with a resolution of 4 cm^−1^, and 64 scans were co-added and averaged to improve the signal-to-noise ratio. The resulting spectra were displayed as absorbance spectra.

##### Data Analysis

For spectral pre-processing, infrared data were converted to ASCII format by OPUS reader software prior to statistical analysis, and imported into Matlab version 2012 (MathWorks, Natick, MA, USA). To minimize problems arising from baseline shifts, the initial step was to remove atmospheric CO_2_ vibrations in the area of 2400–2275 cm^−1^ and replace this with a trend, and the spectra were then normalised using extended multiplicative signal correction (EMSC) [42].

For cluster analysis, the unsupervised dimension reduction method of principal component analysis (PCA) [43] was conducted on the spectra in order to reduce the dimensionality of the multivariate data whilst preserving the variance, prior to the supervised clustering method discriminant function analysis (DFA). In PCA, the inputs are clustered without a *priori* knowledge [29]. By contrast, DFA was performed to create a set of discriminant functions (DFs) on the basis of the retained principal components (PCs) which minimize *within* class differences whilst maximizing the differences *between* the known groups (classes) [44,45]. PC-DFA was performed using PCs 1-30 and the first three DFs were extracted. The class structure for the DFA algorithm was on the basis of the biological replicates from each sample of the same conditions.

## 3. Results and Discussion

### 3.1. Effect of Toluene on the Growth of *P. putida* DOT-T1E Cells

The ability of *P. putida* DOT-T1E strains to grow on LB medium in the presence of different levels of toluene was examined, and the resultant growth curves of the three strains of DOT-T1E are displayed in Figure S1. It is evident that the growth of *P. putida* DOT-T1E strains were inhibited by the addition of different concentrations of toluene to the growth medium, and that the yield of bacterial cells decreased monotonically with increasing toluene concentrations. We note that in control medium, the wild-type DOT-T1E and DOT-T1E-PS28 had similar growth profiles, but that the DOT-T1E-18 mutant grew a little slower, possibly due to the fact that this strain contained higher levels of toluene compared to either DOT-T1E or DOT-T1E-PS28. Indeed, similar findings were obtained in previous reports which investigated the effects of solvents on bacterial biomass yield and it was deduced that the yields were reduced in the presence of toluene in the culture [46,47,48]. Solvent tolerance is an energy intensive process, thus, a possible suggestion is that the decrease in the yield could be caused by an energy-consuming adaptation mechanism such as efflux pump systems in *P. putida* [49,50,51] being used to protect the cells from further damage. It was reported that the biomass yield of *P. putida* S12 and DOT-T1E were reduced when grown in the presence of solvents, suggesting that solvent tolerance demand high levels of energy to cope with the solvent stress [8,52].

In Gram-negative bacteria, efflux pumps are considered to be the most important adaptation mechanism for solvent tolerance [15]. Several studies have demonstrated that an energy-dependent efflux system is responsible for the resistance to toluene in *P. putida* DOT-T1, DOT-T1E and S12 [11,48,49]. To evaluate the role of efflux pump systems in toluene tolerance, growth of the parent strain was directly compared with that of the mutant strains, both in the presence and absence of toluene. The growth of the wild-type was inhibited at all tested concentrations (0.1%, 0.5%, 1.0%, 5.0% (*v/v*) toluene), while the mutants could not grow in the media containing equal to or greater than 1% (*v*/*v*) toluene. Obviously, the mutants were found to be more sensitive to toluene than DOT-T1E, suggesting that TtgABC and TtgGHI pumps play an important role in toluene extrusion.

### 3.2. The Effect of Divalent Cations on the Growth of P. putida DOT-T1E Cultures in the Absence and Presence of Toluene

**Figure 1:**
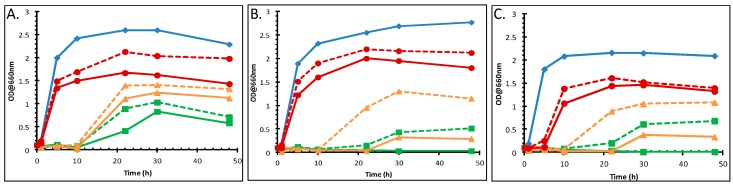
Influence of 7 mM MgSO_4_ on growth of *P. putida* DOT-T1E strains in the presence of toluene. Growth curves of: (**A**) the wild-type DOT-T1E; (**B**) the mutant DOT-T1E-PS28; and (**C**) the mutant DOT-T1E-18. Symbols and colours represent different growth conditions. Control cultures with no toluene (blue closed diamonds), exposed cultures to 0.1% (*v*/*v*) toluene (red closed circles), 0.5% (*v*/*v*) toluene (yellow closed triangles), 1% (*v*/*v*) toluene (green closed square). Solid and dotted lines represent the *absence* and *presence* of metal ion in the culture respectively. A 1/10 dilution of 100 µL samples was prepared to determine the turbidity at 660 nm.

Many Gram-negative bacteria are less sensitive to organic solvents upon the addition of cations (most notably Mg^2+^, Ca^2+^) [53]. Therefore, the influence of Mg^2+^ and Ca^2+^ on the stabilization of the toluene tolerance of *P. putida* DOT-T1E cultures was investigated and the resultant growth curves are shown in Figure 1. Growth was observed when Mg^2+^ was added to the LB medium containing toluene, compared with the control with the absence of metal ions. In the presence of the magnesium ions, the lag period was found to be shorter, and higher cell biomass yields were obtained. The mutants were unable to grow in the presence of toluene at 1% (*v*/*v*) without the metal ion. However, it was observed that the addition of Mg^2+^ improved toluene solvent tolerance and that cultures grew after 20 h incubation time. The effect of the addition of various concentrations of magnesium ions was also tested. Growth increased in the presence of metal ions and 3.5 mM Mg^2+^ was as effective for solvent tolerance as 30 mM (Figure S2). One study showed the influence of various combined concentrations of Ca^2+^ and Mg^2+^ ions on the growth of *P. putida* IH-2000, where growth was improved by the addition of more than 0.5 mM Ca^2+^ and in the presence of more than 2 mM Mg^2+^ [12].

To test the role of metallic cations and anions on the stability of the toluene tolerance, growth was determined after incubation for 24 h at 30 °C in LB containing MgSO_4_, MgCl_2_, Ca(NO_3_)_2_, and CaCl_2_ at 7 mM for Mg^2+^ and 3 mM for Ca^2+^ (other concentrations of these two cations were tested and these were found to be the most optimal (data not shown)) supplemented without or with toluene at 5% (*v*/*v*). The reason that 5% (*v*/*v*) was chosen rather than the lower toluene concentrations (0.5%, 1%) was that in preliminary experiments the most striking effect on bacterial growth/toluene tolerance was seen at 5% beyond 10 h of solvent exposure (data not shown). The *P. putida* DOT-T1E strain has a higher tolerance for toluene in the presence of metal ions and the lag phase period was also significantly shorter (Figure S3). These divalent cations certainly exert beneficial effects as determined by higher cell yield in the presence and absence of toluene. Addition of Mg^2+^ was found to be slightly more effective than Ca^2+^ in improving solvent tolerance in *P. putida* DOT-T1E cells at 22 h time point. With different anions, such as Cl^−^, SO_4_^2−^ and NO_3_^−^, we found that similar growth patterns were obtained under the same culture conditions although the cultures were supplemented with different anions. These observations suggest that the cations were more effective than anions, or that anions may not play a crucial role for stability of solvent tolerance in *P. putida* strains. These results were in agreement with previous observations that Mg^2+^ and Ca^2+^ ions are important for bacterial solvent tolerance [8,12,53].

### 3.3. FT-IR Fingerprinting of *P. putida* DOT-T1E Cultures

In recent years, much attention in the literature has been paid to investigating stress responses in bacteria via the application of metabolomics-based methods [54,55,56], an area which has been applied to a broad range of disciplines including medical sciences, metabolic engineering and drug discovery [57,58,59,60].

**Figure 2:**
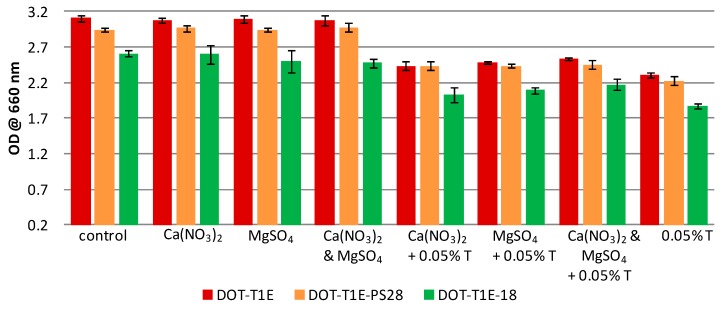
Turbidity at OD 660 nm of *P. putida* strains after 8 h incubation in LB medium supplemented with or without 7 mM magnesium and 3 mM calcium in the absence and presence of 0.05% (*v*/*v*) toluene. Colours represent different strains: the wild-type DOT-T1E (red), the mutant DOT-T1E-PS28 (yellow), and the mutant DOT-T1E-18 (green). Bars of the means of four replicates and error bars are standard deviations.

In this study, a metabolic fingerprinting approach [33] based on FT-IR spectroscopy [61] was employed to study the influence of metal ions on the whole-organism phenotype of *P. putida* DOT-T1E strains in the presence and absence of toluene. To ensure that there was sufficient biomass for metabolomics analysis, cultures were grown in LB medium supplemented without or with 7 mM magnesium and 3 mM calcium in the absence/presence of 0.05% (*v*/*v*) toluene and incubated for 8 h (see Figure 2). The results showed that all strains have the ability to grow in the absence/presence of toluene; however, all *P. putida* strains had higher tolerance to toluene in the presence of divalent cations. By contrast, similar growth profiles were observed in the culture medium supplemented with or without divalent cations in the absence of toluene.

**Figure 3:**
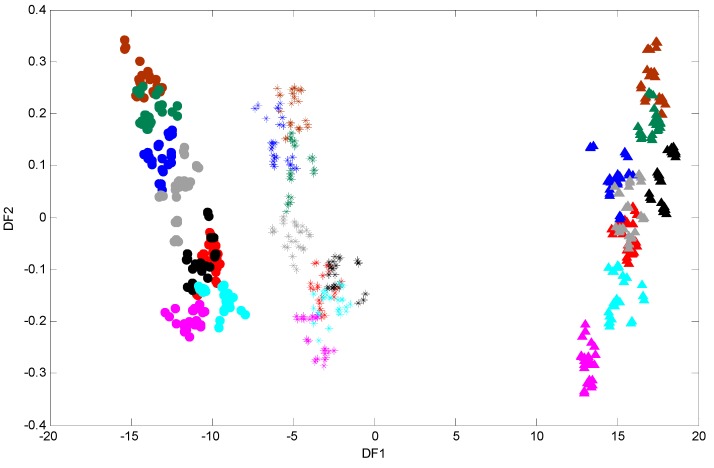
PC-DFA scores plots of FT-IR data of *P.* *putida* DOT-T1E strains in LB medium supplemented with or without 7 mM magnesium and 3 mM calcium in the absence and presence of 0.05% (*v/v*) toluene. Symbols represent different strains: *P. putida* DOT-T1E wild-type (stars), *P. putida* DOT-T1E-PS28 (closed circles), and *P. putida* DOT-T1E-18 (closed triangles). PCs 1-30 with a total explained variance (TEV) of 99.92% were used for the DFA. Colour coding: control with no toluene (red), cells without toluene in the presence of 7 mM Mg^2+^ (brown), 3 mM Ca^2+^ (black), mixed 7 mM Mg^2+^ and 3 mM Ca^2+^ (green), cells challenged with 0.05% (*v/v*) toluene in the presence of 7 mM Mg^2+^ (dark blue), 3 mM Ca^2+^ (light blue), mixed 7 mM Mg^2+^ and 3 mM Ca^2+^ (grey), and cells with 0.05% (*v/v*) toluene in the absence of divalent cations (pink).

To generate robust biochemical fingerprints of *P. putida* DOT-T1E strains, FT-IR spectroscopy was employed. Since subtle and important variations in FT-IR spectra are not easy to interpret visually (Figure S4), chemometric methods were conducted in order to analyse these data in far more detail. Initially, a PCA scores plot was produced (data not shown) and no obvious clusters were observed in this analysis. PCA failed to discriminate data as in many previous studies [44,62]. Therefore, it would seem sensible to employ a supervised clustering approach such as DFA in order to visualise the distribution of samples based on their IR metabolic fingerprint [34]. The first and second discriminant function (DF) scores were generated to identify variation or relationships between the samples, and the resultant PC-DFA scores plot of DF1 *vs.* DF2 is displayed in (Figure 3). As can be seen in Figure 3, a clear separation between the wild-type DOT-T1E and the mutants DOT-T1E-PS28 and DOT-T1E-18 is observed in the first discriminant function which explains the majority of the total group variance (here the groups relate to the biological replicates and are not biased based on either the level of toluene or the addition of cations). This observation could be due to the lack of efflux pump in the mutants compared to the parent strain or an indirect effect on growth of mutant DOT-T1E-18, indicating the ability of FT-IR to discriminate between bacterial cells within the same strain. Figure 3 also clearly shows that a similar trend (through DF2) was observed between the wild-type and the mutants under the same conditions, indicating clear metabolic changes caused by metal ions in the absence and presence of toluene. The parent and the mutant strains have the same genetic background and the only difference between the three cell types is the absence of one of the efflux pump proteins in the mutants compared with the parent strain. Therefore, the results from DFA would suggest that the influence of Mg^2+^ and Ca^2+^ on the stabilization of the toluene tolerance of *P. putida* DOT-T1E may be due to the contribution of metal ions in other bacterial-tolerance mechanisms rather than only the efflux pump(s).

In addition, cells exposed to 0.05% (*v*/*v*) toluene in the absence of metal ions in the wild-type *P. putida* DOT-T1E and the mutant *P. putida* DOT-T1E-PS28 (a mutant in the TtgGHI pump) are clustered more closely to the control cultures compared with the mutant *P. putida* DOT-T1E-18 (which lacks the TtgABC pump), indicating that DOT-T1E-18 cells were more sensitive to 0.05% (*v*/*v*) toluene compared to DOT-T1E and DOT-T1E-PS28 cells. This clustering pattern would suggest that the TtgABC pump might play a more crucial role in toluene efflux than the TtgGHI pump. This observation was in agreement with previous investigations which conclude that the TtgABC pump is the main extrusion pump, and is able to extrude solvents and antibiotics [63,64,65]. Therefore, the results from DFA clearly illustrate that the metabolic fingerprinting approach has the ability to detect a clear effect upon the cell cultures caused by metal ions and toluene which may cause changes to the phenotype of cells.

**Figure 4:**
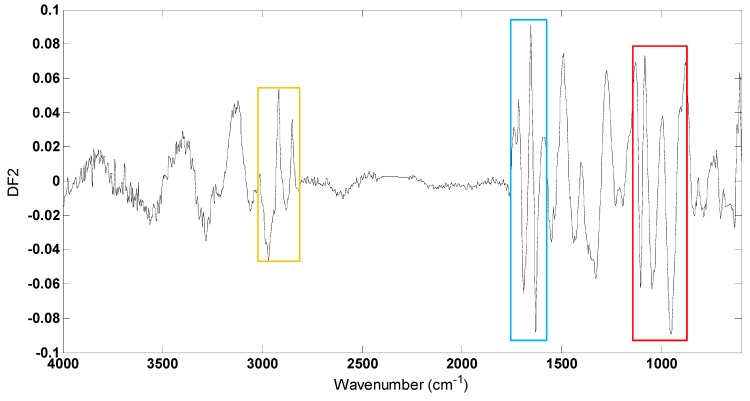
PC-DFA loadings plot from DF 2 of *P. putida* DOT-T1E strains in LB medium supplemented with or without 7 mM magnesium and 3 mM calcium in the absence and presence of 0.05% (*v/v*) toluene. Significant loadings were assigned to bacterial lipids (highlighted in the yellow box), proteins (blue box) and polysaccharides (red box).

To investigate which spectral regions discriminated between different conditions within strains, DFA loadings vectors were calculated and plotted for DF2 (Figure 4) which largely discriminated between different conditions (Figure 3). Several changes occur within these loading plots with the greatest variances being observed between 2950–2850 cm^−1^, 1700–1600 cm^−1^ and 1110–945 cm^−1^ contributed to the DFA score plot clustering. Vibrational assignments are provided in Table 1; in this region of mid-infrared, the bands at 2918 cm^−1^ and 2853 cm^−1^ can be attributed to C-H stretching vibrations from membrane lipids and the peaks at 1630 cm^−1^ and 1550 cm^−1^ would be attributed to C=O stretching (amide I) and a combination of C-N stretching and N-H bending (amide II) vibrations, respectively, from protein components. In addition, the bands at 1105 cm^−1^ and 952 cm^−1^ could arise from a range of vibrations from the carbohydrates family including complex polysaccharide within the cells. These large variations in lipids, proteins and carbohydrates between different conditions within the *P. putida* DOT-T1E cells are due to the biological effects caused by the metal ions and toluene.

The outer membrane of Gram-negative bacteria is an effective barrier for many toxic agents, and divalent cations (in particular, Mg^2+^ and Ca^2+^) are important in the organisation of the outer membrane [67] as lipopolysaccharide (LPS) molecules are linked to each other electrostatically via divalent cations [68,69]. In several cases it has been observed that when the structure of the outer membrane of certain organisms (which are able to acquire resistance against toxic solvents (e.g., toluene)), are modified by chemical or enzymatic removal of parts of the LPS molecule or mutation, the resistance of these bacteria to these solvents is decreased [70,71,72]. On the other hand, Junker *et al.* (2001) observed that in a WbpL mutant of *P. putida* DOT-T1E, LPS may not be important for aromatic hydrocarbon tolerance [73]. If Mg^2+^ and Ca^2+^ are essential for the integrity of the outer membrane and LPS layer, the presence of many aromatic hydrocarbons (e.g., toluene), ethylenediaminetetraacetic acid (EDTA) and antimicrobial peptides (AMPs), lead to significant changes in the structure and function of membrane components, such as disruption and removal of lipids and proteins as well as loss of Mg^2+^ and Ca^2+^ [2,74,75,76].

In Gram-negative bacteria, Clifton *et al.* [77] reported that the removal of calcium ions from the LPS bilayer led to the destabilisation of the bilayer and mixing of LPS molecules between the inner and outer leaflets; indicating the important role of salt bridges which are formed by divalent cations (e.g., Mg^2+^ and Ca^2+^) with negatively charged sugar in LPS core oligosaccharide to strengthen the integrity of the outer membrane. It has been found that calcium has the ability to block the binding of a cationic antimicrobial peptide to LPS and thus decrease its antimicrobial activity [78]. The effect of AMPs, EDTA and Mg^2+^ on the LPS layer was examined in Gram-negative bacteria [77], showing that cationic AMPs or anionic EDTA effectively modify the LPS layer electrostatically by displacing Mg^2+^ ions from the LPS layer competitively, while Mg^2+^ tightens and stabilises the LPS layer [79].

Therefore, it is perhaps not surprising that a similar trend in the DFA scores plot between the wild-type and the mutants were observed, suggesting the contribution of Mg^2+^ and Ca^2+^ in LPS stabilisation but not efflux pumps. This observation would suggest that the efflux pumps system in *P. putida* might not require a magnesium or calcium gradient to export substrates such as toluene. In addition, the most significant changes observed from the interpretation of FT-IR spectra were in the vibration frequency of the polysaccharide, protein and lipid components, and we can infer from this that the important role of divalent cations in *P. putida* DOT-T1E strain is related to LPS mechanism to cope with the presence of toluene.

**Figure 5:**
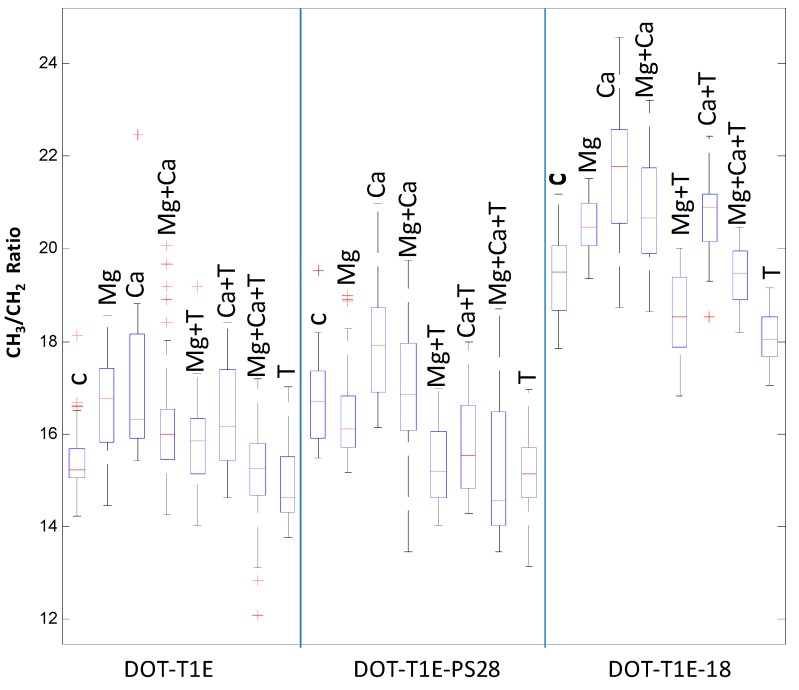
Box-whisker plot for FT-IR scaled spectra showing the ratio of saturated fatty acids (CH_3_:CH_2_) of *P. putida* DOT-T1E strains grown strains in LB medium supplemented with or without 7 mM magnesium and 3 mM calcium in the absence and presence of 0.05% (*v*/*v*) toluene. Red lines indicate the median of peak area of saturated fatty acid ratio of infrared spectra. The median was used to compare the level of saturated fatty acid ratio. Red plus signs represent the outliers.

Finally, the ratio of saturated fatty acid composition was calculated from the raw (Figure 5) and scaled infrared spectra (Figure S5) to investigate the effect of divalent cations and toluene on *P. putida* DOT-T1E strains. It is clear that upon toluene exposure, the saturated fatty acid ratio (CH_3_:CH_2_) was lower compared to the control cultures in the absence of these divalent cations. This result is in agreement with previous observations showing that the fluidity of *P. putida* S12 outer membrane increased in the presence of toluene, as toluene may displace divalent cations from the LPS layer, causing increased membrane permeability [80]. By contrast, the saturated fatty acid ratio of *P. putida* cells was increased with the addition of Mg^2+^ and Ca^2+^ to medium with and without toluene. However, under the same conditions there was a slight decrease in the saturated fatty acid ratio for *P. putida* DOT-T1E-PS28 in the presence of Mg^2+^. In *Pseudomonas aeruginosa*, Schneck *et al.* [81] were able to show that the conformation of the O-antigen was shorter and had a denser layer in the presence of Ca^2+^ compared to the absence of calcium ions. Our results would suggest that divalent cations are essential for the integrity of the LPS layer and the outer membrane and therefore they may play an important role to improve solvent tolerance in *P. putida* cells.

## 4. Conclusions

In this study we have shown that different levels of toluene inhibit the growth and reduce the biomass yields of *P. putida* DOT-T1E strains, suggesting that solvent tolerance demands high levels of energy to cope with toluene stress. In addition, our results clearly show how divalent cations improve toluene tolerance in *P. putida* cells, indicating that Mg^2+^ and Ca^2+^ ions are important for bacterial solvent tolerance. We report that results of PC-DFA from metabolic fingerprinting show obvious separation between different culture conditions and the DFA loadings vectors reveal that several mid-infrared regions derived from lipids, proteins and polysaccharides contribute to this separation. Since results from PC-DFA obtained from the wild-type strain show a very similar trend to that of the mutant cells, it is clearly demonstrated that the influence of divalent cations to improve toluene tolerance in *P. putida* cells may be correlated to other bacterial-tolerance mechanisms including lipopolysaccharides (LPS), but they do not contribute to efflux pumps. Furthermore, divalent cations increase the saturated fatty acid ratio of *P. putida* cells, indicating that Mg^2+^ and Ca^2+^ would be essential for the integrity of the LPS layer and the outer membrane and therefore improve solvent tolerance in bacterial cells.

In conclusion, we have demonstrated that metabolic fingerprinting with appropriate chemometric analysis is a valuable approach for studying the influence of divalent cations on the stabilization of the toluene tolerance of *P. putida* DOT-T1E cultures, advancing our understanding of the role of metal ions in these environmentally and industrially important bacterial cells.

## Figures and Tables

**Table 1:** Wavenumber regions of biological interest and assignment for *P. putida* DOT-T1E cells [66].

Wavenumbers (cm^−1^)	Assignment	FT-IR Vibrational Modes
Lipid		
(2958–2873)	Membrane lipid	Asymmetric CH_3_ stretches mode of CH_3_ end groups from membrane lipid
(2924–2850)	Membrane lipid	Symmetric CH_2_ stretches mode of CH_2_ chain from membrane lipid
Protein		
(3400–3300)	Amide A	N-H stretching
(1690–1620)	Amide I	C=O stretching
(1590–1530)	Amide II	C-N stretching and N-H bending
(1450–1200)		COOH of proteins, free amino acids, polysaccharides
Carbohydrate		
(1200–900)	Polysaccharides	C-O or O-H stretching from polysaccharides

## Supplementary Materials

Supplementary File 1
