# Supplementary material for: Metabolic Fingerprinting of Pseudomonas putida DOT-T1E Strains: Understanding the Influence of Divalent Cations in Adaptation Mechanisms Following Exposure to Toluene
Source: Metabolites. 2016 Apr 26;6(2):14. doi: 10.3390/metabo6020014 (PMC4931545; doi:10.3390/metabo6020014)
Supplement: Supplementary File 1 [file metabolites-06-00014-s001.pdf]

Supplementary Information

Metabolic fingerprinting of *Pseudomonas putida* DOT-T1E strains: understanding the influence of divalent cations in adaptation mechanisms following exposure to toluene

Ali Sayqal, Yun Xu, Drupad K. Trivedi, Najla AlMasoud, David I. Ellis and Royston Goodacre

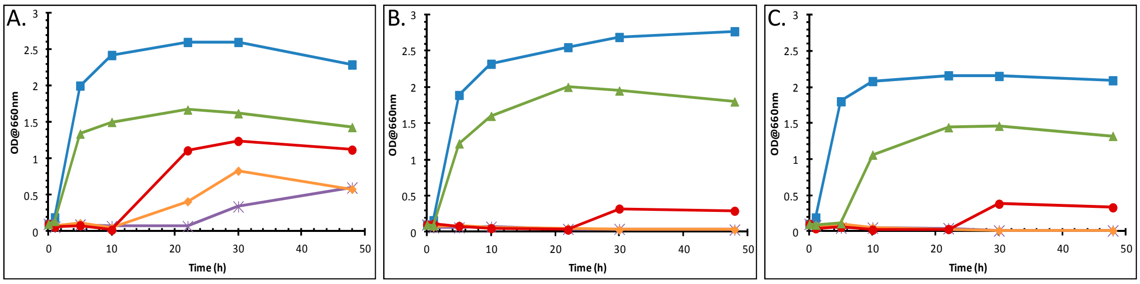

Fig. S1: Growth of *P. putida* DOT-T1E strains on toluene at four different concentrations. Growth curves of: (A) the wild-type DOT-T1E, (B) the mutant DOT-T1E-PS28, and (C) the mutant DOT-T1E-18. Symbols and colours represent different concentration of toluene. Control cultures - no toluene (blue closed squares), exposed cultures to 0.1% (*v/v*) toluene (green closed triangles), 0.5% (*v/v*) toluene (red closed circles), 1% (*v/v*) toluene (yellow closed diamonds) and 5% (*v/v*) toluene (purple stars). A 1/10 dilution of 100 µL samples was prepared to determine the turbidity at 660 nm.

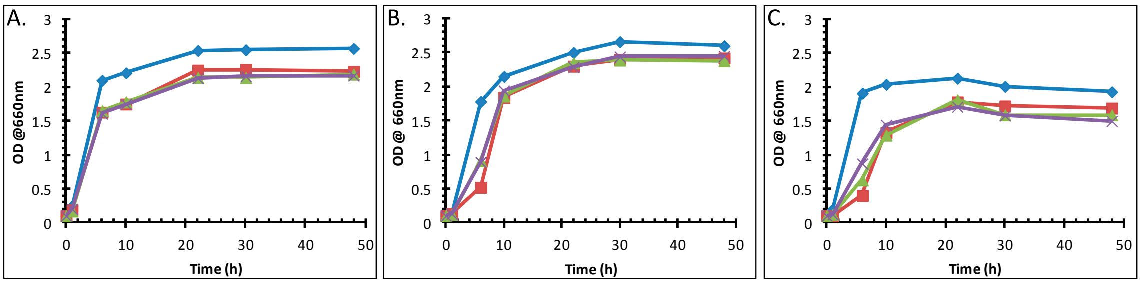

Fig. S2: Influence of three different concentrations of magnesium ion on growth of *P. putida* DOT-T1E strains in the presence of 0.1% (*v/v*) toluene. Growth curves of: (A) DOT-T1E, (B) DOT-T1E-PS28 and (C) DOT-T1E-18. Symbols and colours represent different concentration of metal ion. Control cultures - no toluene and metal ion (blue closed diamonds),exposed cells to 0.1% (*v/v*) in the presence of 3.5 mM Mg^{2+} (red closed squares), 14 mM Mg^{2+} (green closed triangles), 30 mM Mg^{2+} (purple crosses). A 1/10 dilution of 100 µL samples was prepared to determine the turbidity at 660 nm.

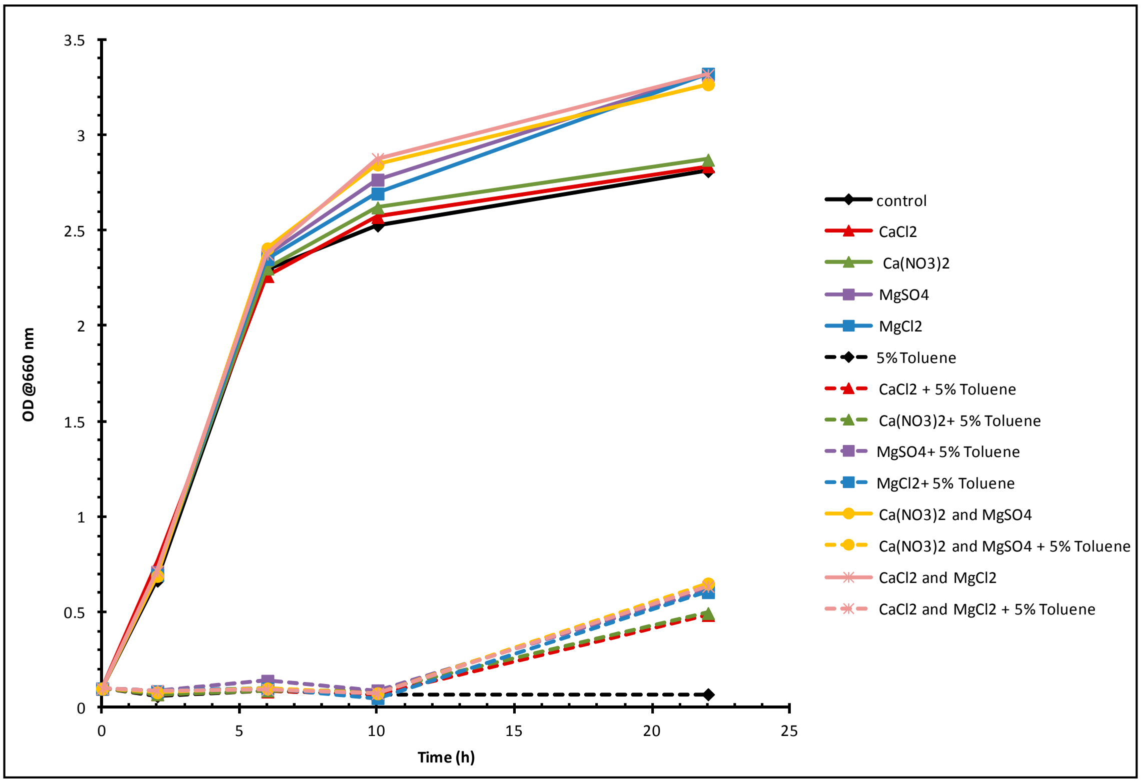

Fig. S3: The influence of cations and anions of metal ions on the growth of *P. putida* DOT-T1E strain in the *absence* and presence of 5% (*v/v*) toluene. Symbols and colours represent different growth conditions. Solid lines represent the *absence* of toluene in the culture, while dotted lines represent the presence of toluene in the culture. A 1/10 dilution of 100 μL samples was prepared to determine the turbidity at 660 nm.

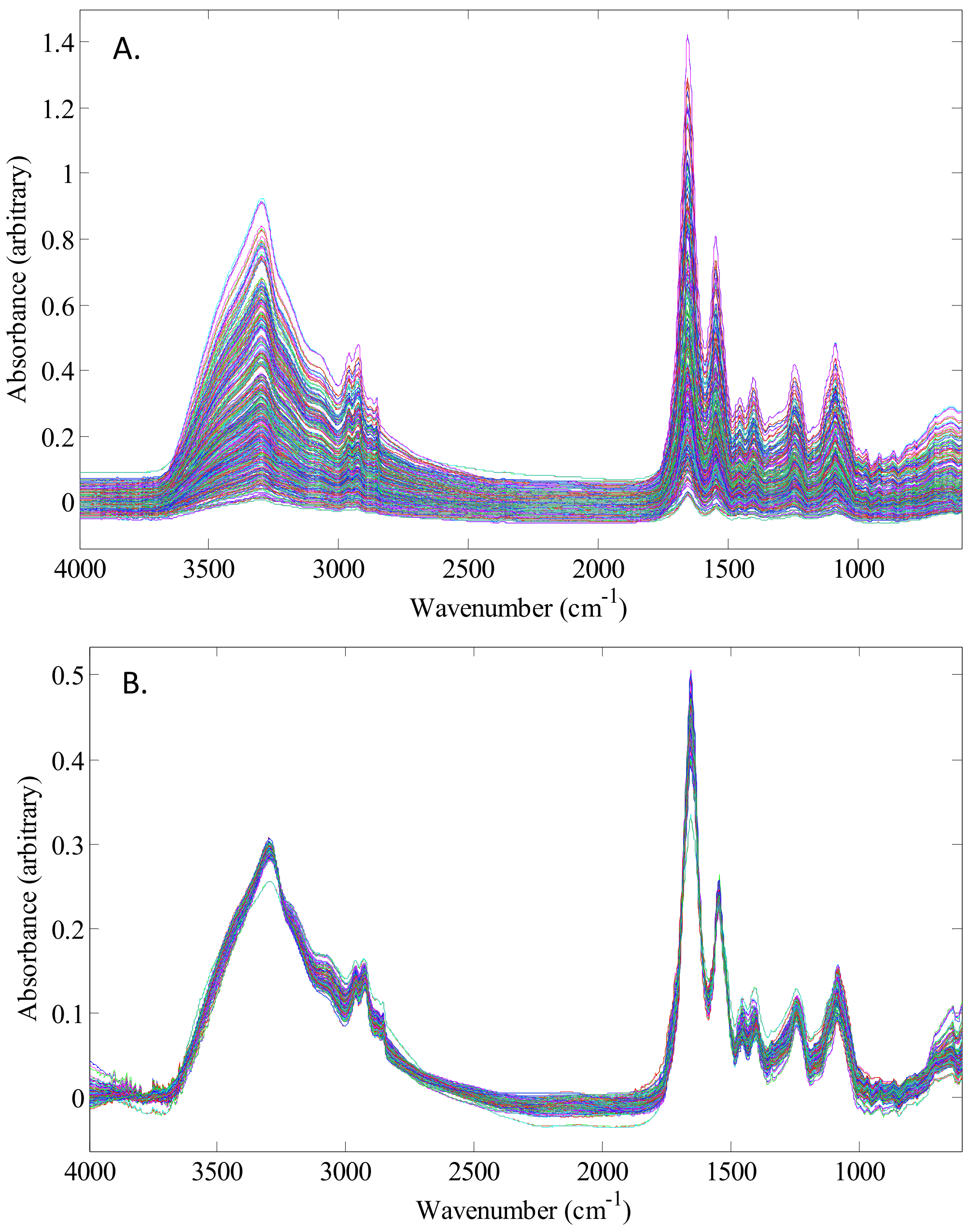

Fig. S4: FT-IR spectra collected for *P. putida* DOT-T1E cultures in LB medium supplemented with or without 7 mM magnesium and 3 mM calcium in the *absence* and presence of 0.05% (*v/v*) toluene. (A) FT-IR raw spectra, while (B) scaled spectra using extended multiplicative signal correction (EMSC).

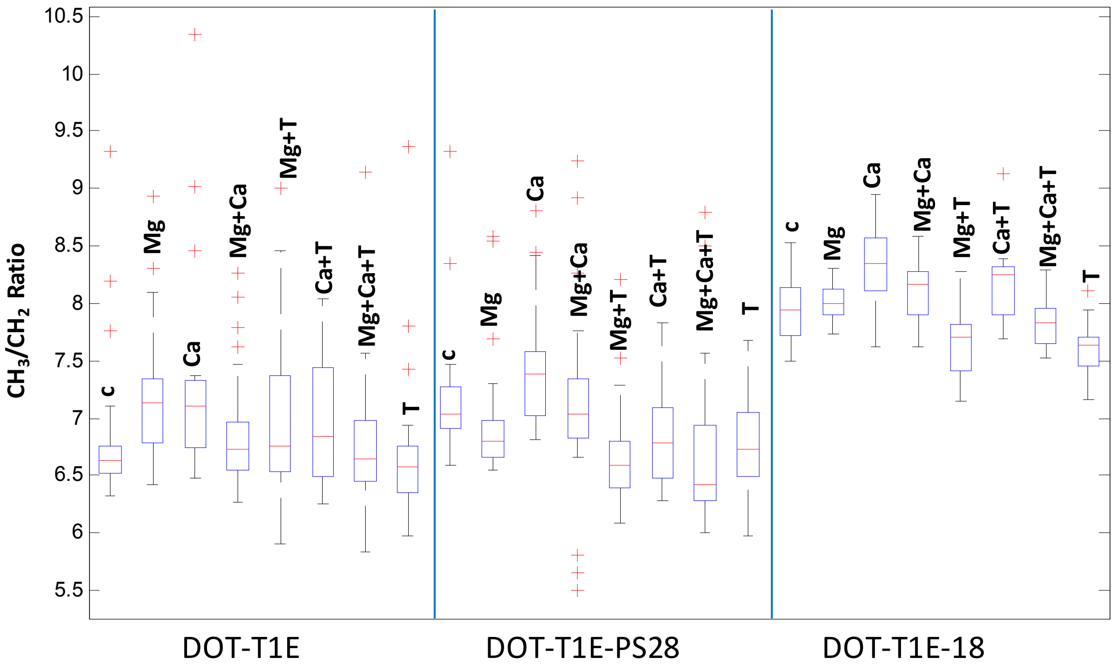

Fig. S5: Box-whisker plot for FT-IR raw spectra showing the ratio of saturated fatty acids (CH_3:CH_2) of *P. putida* DOT-T1E strains grown in LB medium supplemented with or without 7 mM magnesium and 3 mM calcium in the *absence* and presence of 0.05% (*v/v*) toluene. The red lines indicate the median of peak area of saturated fatty acids ratio of infrared spectra. The median was used to compare the level of saturated fatty acids ratio. Red plus signs represent outliers.
